# The clinical & neurophysiological study of leprosy

**DOI:** 10.12669/pjms.303.5354

**Published:** 2014

**Authors:** Murat Cabalar, Vildan Yayla, Samiye Ulutas, Songul Senadim, Ayla Culha Oktar

**Affiliations:** 1 Dr. Murat Cabalar, Department of Neurology, Bakirkoy Dr. Sadi Konuk Research & Training Hospital, Istanbul, Turkey.; 2 Dr. Vildan Yayla, Department of Neurology, Bakirkoy Dr. Sadi Konuk Research & Training Hospital, Istanbul, Turkey.; 3 Dr. Samiye Ulutas, Department of Neurology, Bakirkoy Dr. Sadi Konuk Research & Training Hospital, Istanbul, Turkey.; 4 Dr. Songul Senadim, Department of Neurology, Bakirkoy Dr. Sadi Konuk Research & Training Hospital, Istanbul, Turkey.; 5 Dr. Ayla Culha Oktar, Department of Neurology, Bakirkoy Dr. Sadi Konuk Research & Training Hospital, Istanbul, Turkey.

**Keywords:** Disability, Leprosy, Neuropathy, Neurophysiology

## Abstract

***Objectives:*** The aim of this study was to evaluate neurological and neurophysiological features of leprosy.

***Methods:*** Seventy seven hospitalized leprosy patients (52 male, 25 female) were examined neurological and neurophysiologically between 2010 and 2012. Standard procedures were performed for evaluating sensory and motor conduction studies to all patients. Motor studies were carried out on median, ulnar, tibial and common peroneal nerves. Sensory studies were carried out on median, ulnar and sural nerves. Sympathetic skin response (SSR) recordings on both hands and feet, and the heart rate (R-R) interval variation (RRIV) recordings on precordial region were done in order to evaluate the autonomic dysfunction.

***Results:*** The mean age was 59.11±14.95 years ranging between 17 and 80 years. The mean duration of disease was 35.58±18.30 years. Clinically, the patients had severe deformity and disability. In neurophysiological examinations, sensory, motor conduction studies of the lower extremities were found to be more severely affected than upper, and sensory impairment predominated over motor. Abnormal SSRs were recorded in 63 (81.8%) cases of leprosy. Abnormal RRIVs were recorded in 41 (53.2%) cases and abnormal RRIVs with hyperventilation were recorded in 55 (71.4%) cases of leprosy. Significant differences were found between SSR and sensory conduction parameters of median, ulnar nerves as well as motor conduction parameters of median, ulnar and peroneal nerves (p<0.05).

***Conclusion: ***Peripheral nervous system dysfunction is accompanied by autonomic nervous system dysfunction in leprosy patients. Sympathetic involvement may predominate over parasympathetic involvement.

## INTRODUCTION

Leprosy is a chronic granulomatous and infectious disease caused by Mycobacterium leprae (ML) affecting the skin, eye, nasal mucosa, testis, kidney, both somatic and autonomic nerves. Secondary complications of the neuropathy can result in deformity and disability. Leprosy affects people of all ages and both sexes. Dermato-neurological signs and symptoms are the primary manifestations, ranging in spectrum between two forms as tuberculoid and lepromatous. The disease is thought to be of low infectivity. Transmission of the ML is through nasal secretions and skin contact, and people at higher risk are those who live in the same house as the carrier of ML.^1^^-^^3^

Clinical examination, focal abnormalities, skin lesions as dry, hairless are common. Electrodiagnostic findings early in the disease reveal demyelinating features, such as slowing of conduction velocity and prolongation of latencies, but as the disease progresses secondary axonal damage commonly ensues.^4^^,^^5^

In this study, after clinical examination, motor and sensory nerve conduction studies, sympathetic skin responses (SSR) and R-R interval variations (RRIV) were performed in chronic leprosy patients. 

## METHODS

This investigation was carried out between 2010 and 2012 on leprosy patients who hospitalized to the Leprosy Clinic of Turkan Saylan in Istanbul. We studied 77 leprosy patients (25 female, 52 male), the mean age was 59.11±14.95 years ranging between 17 and 80 years. The mean duration of disease was 35.58±18.30 years. Routine neurological examinations and neurophysiological evaluation were performed on all patients.

Neurophysiological tests were performed by Medelec Synergy EMG apparatus. Sensory conduction studies of right or if not available left median, ulnar and sural nerves, and motor conduction studies of median, ulnar, peroneal and tibial nerves were obtained by standard procedures. Motor distal latencies (ms), amplitudes of compound motor action potential (CMAP) (mV) and motor conduction velocities (m/s) were evaluated. Onset and peak latencies (ms): and amplitudes (μV) of sensory nerve action potentials (SNAP) and sensory nerve conduction velocities (m/s) were studied. Cup electrodes were used to record SSRs: The active electrode was placed on the palmar surface of the hand, while the reference electrode was placed on the dorsum of the hand and foot if available. The SSRs were recorded by wrist stimulation for the median nerve or malleolar stimulation for tibial nevre. The R-R interval variation and RRIV with hyperventilation studies (RRIV HV) were performed using two cup electrodes placed on the precordial region and results were evaluated according to the formula described by Shahani.^6^

***Data Analysis: ***NCSS (Number Cruncher Statistical System) 2007 Statistical Software (Utah, USA) was used for statistical analyses. Defining statistical methods (mean value, standard deviation and frequency of distribution) are used for evaluation of data. Chi-square test with Yates’ correction and Fisher exact test were used to compare qualitative data. The results were considered statistically significant as p<0.05.

***Ethical Considerations: ***The research was approved by the Research Ethics Committee of the Bakirkoy Dr. Sadi Konuk Resarch and Education Hospital, with protocol number: 2012.06.05, date: 09.04.2012. All subjects gave their written informed consent. 

## RESULTS

Most of the cases (n=48) originated from eastern part of Turkey. Mean hospitalisation time was 32.6±17.9 (7-90) days. Leprosy case in family was reported by 39% (n=30) of individuals, and most of these leprosy cases were first degree relatives (father, mother, and brother).

**Fig.1 (a-b):**
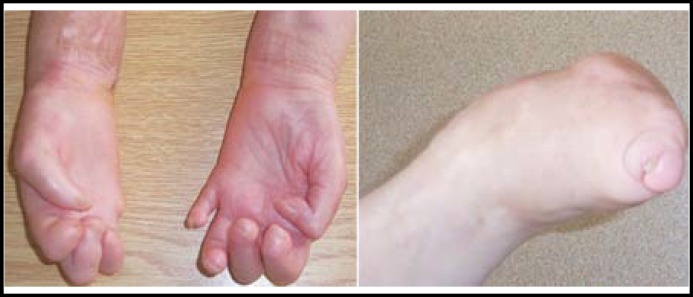
Right hand 5^th^ finger amputee and four fingers of left foot amputee

Clinical examination; Leg, foot or finger amputees of lower extremites were observed in 40 patients, but arm, hand or finger amputees of upper extremites in 18 patients (Fig. 1a and 1). There were lagophthalmos (n=23), ulnar nerve thickening (n=34), sensory loss in glove and stocking pattern in 36 patients, drop hand (n=1), drop foot (n=1) but deep tendon reflexes were preserved in 40 patients. Diabetes mellitus (n=5), visual loss [Cataract (n=28), traumatic (n=6)], hearing loss (n=8), tension headache (n=1), endocrinopathy (n=12) such as osteoporosis, vitamin b12 deficiency, hypothyroidism, and depression (n=3) were other symptoms.

Neurophysiological examination; median, ulnar, tibial and peroneal nerve sensory and motor responses (distal latans, SNAP-CMAP and conduction velocities) are shown in Table I and II. Sural nerve potentials were recorded only in four cases. The parameters were given as min-max and mean±SD consequently: Right sural nerve (n=3) distal latans (ms) (1.9-4.3), (2.96±1.22), SNAP (μV) (3.2-15.3), (7.30±6.93), conduction velocity (m/s) (32.6-73.7) (52.83±20.56) and left sural nerve (n=1) distal latans=7 ms, SNAP=7.8 μV, conduction velocity=20 ms were recorded. Abnormal SSR were recorded in 63 (81.8%) cases of leprosy. Abnormal RRIV were recorded in 41 (53.2%) cases and abnormal RRIV HV were recorded in 55 cases (71.4%) cases of leprosy. In 23 (42.59%) cases who had amputation of foot fingers, SSR were recorded abnormal (p=0.002). In 14 (35%) cases who had amputation of hand fingers, SSR were recorded abnormal (p=0.144). Cases whom detected lagophtalmos (p=0.153, p=0.074, p=0.195 respectively) and sensory loss in glove and stocking pattern (p=0.160, p=0.499, p=0.922 respectively) did not show any statistical difference for SSR, RRIV, and RRIV HV.

Moreover, significant difference was not found between other clinical findings (hypertension, diabetes mellitus, hearing loss, drop hand, and drop foot) and neurophysiological findings (SSR, RRIV, and HV). Significant difference was found between ulnar nerve sensory conduction studies and RRIV (p=0.014). Furthermore, significant differences were found between SSR and median, ulnar sensory conduction studies (p=0.007, p=0.001 respectively) and also median, ulnar, peroneal motor conduction studies (p<0.005, p<0.05, p<0.05 respectively). But, significant differences were not found between other sensory, motor conduction studies and SSR, RRIV, RRIV HV findings. The distrubution of sensory and motor responses in patients with abnormal SSR, RRIV and RRIV HV are shown on Table -III-IV.

**Table-I:** Median and unlar nerves sensory and motor conduction findings

		*Right*		*Left*	
		*Sensory* *min-max * *mean±sd*	*Motor * *min-max * *mean±sd*	*Sensory* *min-max * *mean±sd*	*Motor* *min-max * *mean±sd *
Median Nerve	DL	n=20 1.9-5.2 3.21±0.85	n=34 3-11.4 5.06±1.60	n=5 2-4 2.56±0.82	n=7 2.7-8.8 4.69±2.09
	SNAP CMAP	n=20 4.1-22.1 9.68±5.13	n=34 0.2-10.3 5.31±2.78	n=5 2.9-20.5 9.34±7.30	n=7 0.5-10.4 5.81±3.38
	CV	n=20 23.1-59.5 42.53±10.81	n=33 23-67 48.66±9.68	n=5 37-70 50.50±13.50	n=7 43.7-73 50.64±10.32
	DL	n=12 1.7-4.8 2.94±1.04	n=28 2.5-10 4.16±1.56	n=4 1.6-3.55 2.40±0.84	n=4 2.1-4.56 3.01±1.07
Ulnar Nerve	SNAP CMAP	n=12 4.2-12.9 6.91±2.68	n=27 0-12 5.43±2.98	n=4 9.9-15.4 11.78±2.57	n=4 3.38-7.2 6.00±1.58
	CV	n=12 19.8-57.9 39.01±13.02	n=24 35.7-78.4 55.13±11.47	n=4 31-55 44.20±10.37	n=3 45-55 51.37±5.51
				

DL: distal latency (ms), CV: conduction velocity (m/s),

SNAP: sensory nerve action potential (μV), CMAP: compound motor action potential (mV).

**Table-II:** Tibial and common peroneal nerve motor conduction findings

	*Right*		*Left*	
	*Common peroneal * *nerve* *min-max * *mean±sd*	*Tibial * *nevre* *min-max * *mean±sd*	*Common peroneal * *nerve* *min-max * *mean±sd*	*Tibial * *nerve* *min-max * *mean±sd *
DL	n=20 2.7-12.4 5.17±2.47	n=18 3.75-9.35 6.06±1.52	n=3 2.6-4.95 3.85±1.18	n=3 3.5-8.5 5.42±2.70
CMAP	n=20 0.3-4.9 1.61±1.27	n=18 0.3-7.3 3.07±2.10	n=3 0.4-5.4 2.54±2.57	n=3 0.1-5.3 2.01±2.88
CV	n=18 23-57.1 41.26±8.76	n=17 34.1-52.1 41.09±5.14	n=3 39.3-51.6 46.80±6.66	n=4 27.5-44.2 35.10±8.45

DL: distal latency (ms), CV:conduction velocity (m/s), SNAP:sensory nerve action potential (μV), CMAP:compound motor action potential (mV).

**Table-III:** Distrubution of sensory and motor nerve impairment in patients with abnormal RRIV

	*Sensory*				*Motor*			
	*RRIV normal*	*RRIV abnormal*	*Total*	*p*	*RRIV normal*	*RRIV abnormal*	*Total*	*p*
Median nerve DL SNAP/CMAP CV	14(35%) 14(35%) 14(35%)	7(43.8%) 7(43.8%) 7(43.8%)	21(37.5%) 21(37.5%) 21(37.5%)	0.541 0.541 0.541	27(65.9%) 27(65.9%) 26(65%)	8(50%) 8(50%) 8(50%)	35(61.4%) 35(61.4%) 34(60.7%)	0.269 0.269 0.299
Ulnar nerve DL SNAP/CMAP CV	6(15%) 6(15%) 6(15%)	7(46.7%) 7(46.7%) 7(46.7%)	13(23.6%) 13(23.6%) 13(23.6%)	0.014 0.014 0.014	20(51.3%) 19(50%) 17(47.2%)	8(50%) 8(50%) 8(50%)	28(50.9%) 27(50%) 25(48.1%)	0.931 0.999 0.853
Tibial nerve DL CMAP CV	_	_	_	_	16(59.3%) 16(59.3%) 15(57.5%)	3(33.3%) 3(33.3%) 3(33.3%)	19(52.8%) 19(52.8%) 18(51.4%)	0.117 0.117 0.208
Peroneal nerve DL CMAP CV	_	_	_	_	15(55.6%) 15(55.6%) 13(52%)	4(44.4%) 4(44.4%) 4(44.4%)	19(52.8%) 19(52.8%) 17(50%)	0.563 0.563 0.697
Sural nerve DL SNAP CV	2(6.9%) 1(3.6%) 1(3.6%)	2(20%) 2(20%) 2(20%)	4(10.3%) 3(7.9%) 3(7.9%)	0.239 0.098 0.098	_	_	_	_

DL: distal latency (ms), CV: conduction velocity (m/s),

SNAP: sensory nerve action potential (μV), CMAP: compound motor action potential (mV).

**Table-IV:** Distrubution of sensory and motor nerve impairment in patients with abnormal RRIV HV

	*Sensory*				*Motor*			
	*RRIV HV normal*	*RRIV HV abnormal*	*Total*	*p*	*RRIV HV* *Normal*	*RRIV HV abnormal*	*Total*	*p*
Median nerve DL SNAP/CMAP CV	9(36%) 9(36%) 9(36%)	12(38.7%) 12(38.7%) 12(38.7%)	21(37.5%) 21(37.5%) 21(37.5%)	0.835 0.835 0.835	18(72%) 18(72%) 18(72%)	17(53.1%) 17(53.1%) 16(51.6%)	35(61.4%) 35(61.4%) 34(60.7%)	0.146 0.146 0.120
Ulnar nerve DL SNAP/CMAP CV	5(20%) 5(20%) 5(20%)	8(26.7%) 8(26.7%) 8(26.7%)	13(23.6%) 13(23.6%) 13(23.6%)	0.562 0.562 0.562	15(60%) 14(58.3%) 14(58.3%)	13(43.3%) 13(43.3%) 11(39.3%)	28(50.9%) 27(50%) 25(48.1%)	0.218 0.273 0.171
Tibial nerve DL CMAP CV	_	_	_	_	9(60%) 9(60%) 8(57.1%)	10(47.6%) 10(47.6%) 10(47.6%)	19(52.8%) 19(52.8%) 18(51.4%)	0.463 0.463 0.581
Peroneal nerve DL CMAP CV	_	_	_	_	7(46.7%) 7(46.7%) 7(46.7%)	12(57.1%) 12(57.1%) 10(52.6%)	19(52.8%) 19(52.8%) 17(50%)	0.535 0.535 0.730
Sural nerve DL SNAP CV	2(11.8%) 1(6.3%) 1(6.3%)	2(9.10%) 2(9.10%) 2(9.10%)	4(10.3%) 3(7.9%) 3(7.9%)	0.785 0.748 0.748	_	_	_	_

DL: distal latency (ms), CV: conduction velocity (m/s),

SNAP: sensory nerve action potential (μV), CMAP: compound motor action potential (mV).

**Table-V:** Distrubution of sensory and motor nerve impairment in patients with abnormal SSR

	*Sensory*				*Motor*			
	*SSR* *normal*	*SSR* *abnormal*	*Total*	*p*	*SSR* *Normal*	*SSR* *abnormal*	*Total*	*p*
*Median nerve* DL SNAP/CMAP CV	11(61.1%) 11(61.1%) 11(61.1%)	12(25.5%) 12(25.5%) 12(25.5%)	23(35.4%) 23(35.4%) 23(35.4%)	0.007 0.007 0.007	16(84.2%) 16(84.2%) 16(84.2%)	20(43.5%) 20(43.5%) 19(42.2%)	36(55.4%) 36(55.4%) 35(54.7%)	0.003 0.003 0.002
*Ulnar nerve* DL SNAP/CMAP CV	9(50%) 9(50%) 9(50%)	5(11.1%) 5(11.1%) 5(11.1%)	14(22.2%) 14(22.2%) 14(22.2%)	0.001 0.001 0.001	13(72.2%) 12(70.6%) 10(66.7%)	15(33.3%) 15(33.3%) 14(32.6%)	28(44.4%) 27(43.5%) 24(41.4%)	0.005 0.008 0.021
*Tibial nerve* DL CMAP CV	_	_	_	_	9(64.3%) 9(64.3%) 8(61.5%)	10(41.7%) 10(41.7%) 10(41.7%)	19(50%) 19(50%) 18(48.6%)	0.179 0.179 0.248
*Peroneal nerve* DL CMAP CV	_	_	_	_	10(71.4%) 10(71.4%) 10(71.4%)	9(37.5%) 9(37.5%) 8(34.8%)	19(50%) 19(50%) 18(48.6%)	0.044 0.044 0.031
*Sural nerve* DL SNAP CV	2(13.3%) 2(13.3%) 2(13.3%)	3(11.5%) 2(8%) 2(8%)	5(12.2%) 4(10%) 4(10%)	0.866 0.586 0.586	_	_	_	_

DL: distal latency (ms), CV: conduction velocity (m/s), SNAP: sensory nerve action potential (μV), CMAP: compound motor action potential (mV).

## DISCUSSION

According to the World Heath Organization (WHO) in 2000, 1,319,849 leprosy cases were recorded in the world.^7^ There were 181,941 new cases diagnosed and reported to WHO in 2012.^8^ The Ministry of Health of Turkey reported 2353 leprosy cases in 2004, and prevalence rate was 3.21 per 100,000.^9^

Leprosy causes skin lesions and neuropathy. Secondary complications of the neuropathy can result in deformity and disability. In addition to leprosy remains a stigmatizing disease. Therefore, this perception of stigma in patients may cause them to isolate themselves from society. It may cause anxiety, depression, isolation, problems in family relationships and friendships and reduce treatment adherence and recovery chances.^10^^,^^11^

Lagophthalmos usually results in damage of the zygomatic and temporal branches of the facial nerve. It gives rise to exposure keratopathy. Reduced corneal and conjunctival sensation due to involvement of the ophthalmic branch of the trigeminal nerve predisposes to corneal ulceration.^12^ Kim JH et al. detected 9.7% facial nerve involment and lepromatous leprosy (LL) group displayed the significantly lowest prevalence of facial palsy (%6).^13^ In another study, 192 leprosy cases were investigated and lagophthalmus detected in 23%, cataract detected in 14%.^14^ In his study, Awasthi SK et al. detected sensori-neural hearing loss in 6 patients with LL.^15^ Leal AM and Foss NT reported endocrine dysfunctions such as hypogonadism, sterility, and osteoporosis in leprosy in their studies.^16^ We detected lagophthalmus in 29.9%, cataracts in 36.4%, hearing loss in 10.4% (n=8) of the cases. Nerve thickening on palpation in six, sensory loss in stocking-glove pattern in one, asymmetrical sensory loss in the feet in two, and asymmetrical sensory loss in the hands and feet in 25 patients were observed in a study by Soysal A et al. They reported severe extremity amputations in their cases.^17^ Our results were similiar to this study. Since our cases were long term patients with severe disabilities, we did not consider taking a separate control group. Saraya MA et al. reported that leprosy infection may play a role in the pathogenesis of diabetes mellitus and revealed that diabetes mellitus is more frequently seen in lesprosy cases comparing to the control group.^18^ Diabetes mellitus can cause either peripheral or autonomic neuropathy. In our study we detected only 6.5% (n=5) of the cases had diabetes mellitus. Thus, we thought that the neuropathy is the result of leprosy.

Neuropathy is one of the most frequent complications in leprosy patients manifesting as sensory, motor or autonomic deficit. Sensory loss is the earliest and most frequently affected modality, but predominantly motor loss can also occur. Leprosy most commonly affects the posterior tibial nerve causing anesthesia on the soles of the feet followed by the ulnar, median, lateral popliteal, and fasial nerve. Other nerves affected by the disease include the greater auricular, radial, and radial cutaneous nerves.^19^^,^^12^^,^^20^ Ramadan et al. assessed 40 patients, being the ulnar nerve the most frequent damaged and the claw hand the most common disability. All the sensory modalities were affected: superficial and deep sensitivities. However, deep pressure was altered only on late cases. The sensory impairment predominated over the motor.^21^ Jardim et al. studied 19 patients with primary neural leprosy, and clinically they found sensory and motor losses in 78.9%, followed by neural thickening 68.4%.^22^ Ulnar nerve was the most common affected nerve in our study. One radial nerve (drop hand) and one peroneal nerve (drop foot) were also detected. Neural thickening was detected in 44.12% of all cases.

The neurophysiological evaluation is more sensitive than the clinical examination for the detection of nerve impairment, and the presence of abnormalities is frequent, even on nonenlarged nerves. Patients without clinical involvement presented neurophysiological abnormalities in 40% of the cases.^23^^,^^24^ Our cases had serious clinical involvement and severe disability caused by long term sustaining leprosy disease. McKnight J et al. in leprosy patients in northern India, it was found that the commonest and the earliest impairment was reported in sensory nerve conduction of sural nerve.^25^ Kar S et al. reported a more often and early involvement of ulnar nerve.^26^ In our study, only in 4 cases sural nerve sensory response was observed. Ulnar nerve sensory response was lower than median nerve sensory response. Motor responses were absent in most of the cases (median nerve 46.8%, ulnar nerve 58.4%, tibial nerve 72.7%, peroneal nerve 70.1%) similiar to Soysal A et al.’s study. In this study we also investigated autonomic nerve impairment and SSR was absent in 79.3% of the cases. Compared to the controls, the RRIV values of the patients were found to be reduced during both resting and forced deep hyperventilation. In our study, abnormal SSR were recorded in 63 (81.8%) cases of leprosy. Abnormal RRIV was recorded in 41 (53.2%) cases and abnormal RRIV HV was recorded in 55 (71.4%) cases of leprosy.

The RRIV and SSR reveale autonomic nervous system dysfunction. The RRIV assessment parasympathetic function in the distribution of the vagus nerve. Whereas, the SSR assessment sudomotor function controlled by the sympathetic nervous system.^27^^,^^28^ Both the RRIV and the SSR are often affected simultaneously in autonomic dysfunction. But, it is not surprising that this effect does not always occur in paralel. Ulvi H et al. observed that there was significant correlation between RRIV and SSR. But, there was no significant correlation between duration of leprosy. In this study, the mean duration of disease was 16.73 years.^29^ Ramachandran et al. reported association between severity autonomic neuropathy and longer duration of leprosy. In this study, it was shown that parasympathetic system was affected more than sympathetic system.^30^ Philips JC et al investigated the relationship between duration of leprosy disease, type 1 diabetus mellitus and autonomic neuropathy. Patients were divided into four groups according to diabetes duration (<10 years, 11-20 years, 21-30 years and >30 years from group 1 to group 4, respectively) and compared to the age-matched non-diabetic subjects and it was shown that parasympathetic system was affected more than sympathetic system.^31^ In our study, the mean duration of disease was 35.58±18.30 years. We did not evaluate the correlation between the duration of leprosy and autonomic neuropathy because of the long term of the disease. However, autonomic nerve involvement was together with peripheral nerve involvement, and SSR affection was more than RRIV effect.

In conclusion, leprosy is an important disease causing severe physical disability although it has a reduced prevelance. Peripheral and autonomic nerve involvement was frequent and severe in our cases. Different than other studies, SSR involvement was more frequent than RRIV abnormality. Its cause can be due to severity of our leprosy cases and longer diasease duration.
